# Presentation of hemophagocytic lymphohistiocytosis due to a novel MUNC 13–4 mutation masked by partial therapeutic immunosuppression

**DOI:** 10.1186/1546-0096-10-13

**Published:** 2012-05-03

**Authors:** Jackie P-D Garrett, Irene Fung, Jeremy Rupon, Andrea Knight, Melissa Mizesko, Michelle Paessler, Jordan S Orange

**Affiliations:** 1 Division of Allergy and Immunology, The Children’s Hospital of Philadelphia, 3550 Market Street, Philadelphia, PA 19104-4399, USA; 2 Divisions of Hematology and Oncology, The Children’s Hospital of Philadelphia, 34th and Civic Center Boulevard, Philadelphia, PA 19104, USA; 3 Division of Rheumatology, The Children’s Hospital of Philadelphia, 34th and Civic Center Boulevard, Philadelphia, PA 19104, USA; 4 Department of Pathology and Laboratory Medicine, The Children’s Hospital of Philadelphia, 34th and Civic Center Boulevard, University of Pennsylvania School of Medicine, Philadelphia, PA, 19104, USA

**Keywords:** Hemophagocytic lymphohistiocytosis, MUNC 13–4, Macrophage activation syndrome

## Abstract

Hemophagocytic lymphohistiocytosis is a potentially fatal disease characterized by excessive macrophage and lymphocyte activity. Patients can be affected following immune activation after an oncologic, autoimmune or infectious trigger. An associated gene mutation may be found which impairs cytolytic lymphocyte function. We describe a pediatric case of hemophagocytic lymphohistiocytosis with a novel mutation of MUNC 13–4 whose diagnosis was confounded by concurrent immunosuppression. Clinical reassessment for hemophagocytic lymphohistiocytosis is necessary in persistently febrile patients with laboratory derangements in the setting of immunosuppressive agent exposure.

## Background

Hemophagocytic lymphohistiocytosis (HLH) is a clinical syndrome of abnormal immune activation causing excess inflammation. There is increased ectopic migration and proliferation of T cells, tissue infiltration by activated macrophages (histiocytes), hyper-activation of lymphocytes, and prolonged release of pro-inflammatory cytokines [1]. This leads to uncontrolled inflammation manifesting as fever, cytopenias and organ dysfunction. The syndrome is fatal without prompt symptom recognition and treatment.

Primary HLH, also known as familial HLH, is often considered a pediatric disease. Affected individuals have mutations in genes leading to impaired lytic activity of lymphocytes, including NK cells and cytotoxic T lymphocytes [2]. These individuals are at risk of developing HLH. Mutations are inherited in an autosomal recessive pattern, but can also result from de novo mutations, making family history less reliable for diagnosis.

Acquired HLH, also known as secondary HLH, occurs more commonly in adults. Nonetheless, it is also described in children. There are presently no identifiable genetic mutations linked to this phenotype. Currently, secondary HLH is attributed to an abnormal immune response triggered by an infectious, oncologic or autoimmune etiology. As such, other names for secondary HLH include Virus-Associated Hemophagocytic Syndrome and Malignancy-Associated Hemophagocytic Syndrome [3].

The diagnosis of HLH is based on clinical criteria. The most recent diagnostic guidelines were revised in 2004 [4]. HLH is diagnosed if either a molecular diagnosis consistent with HLH is made, or five of the eight following diagnostic criteria are met: 1.) fever; 2.) splenomegaly; 3.) cytopenias affecting at least two of three lineages in the peripheral blood (haemoglobin <90 g/L, platelets <100 × 10^9^/L, or neutrophils <1 × 10^9^/L); 4.) hypertriglyceridemia (fasting triglycerides ≥ 3 mmol/L (≥ 265 mg/dL)) and/or hypofibrinogenemia (fibrinogen ≤ 1.5 mg/dL); 5.) hemophagocytosis in bone marrow, spleen, or lymph nodes (excluding signs of malignancy); 6.) low or absent NK-cell activity; 7.) hyperferritinemia (ferritin >500 μg/L); and 8.) high levels of sIL-2R (sIL-2R ≥ 2400 U/ml).

Here we present a pediatric case of HLH with a novel mutation in MUNC 13–4 whose diagnosis of HLH was confounded by low dose treatment with immunosuppressive agents thereby complicating her clinical picture.

## Case presentation

Our patient is a 3 year-old female who was previously healthy. Four months prior to hospitalization she began experiencing fatigue, recurrent fevers, progressive muscle weakness, and behavioral changes. The week prior to admission, she had decreased urine output, increased abdominal girth and respiratory distress. She was admitted to a community hospital for possible pneumonia. After 3 days, she was transferred to a tertiary center for oncologic assessment. There, her physical examination was concerning for a tender right axillary lymph node and hepatosplenomegaly. A chest radiograph demonstrated diffuse airspace opacities. PCR studies identified both rhinovirus and mycoplasma in nasal pharyngeal swabs. Twenty-four hours after arrival she developed hypoxemic respiratory failure requiring tracheal intubation and mechanical ventilation. This was complicated by an aspiration event and subsequent cardiopulmonary arrest, requiring resuscitation. Multiple subspecialties convened to facilitate an underlying diagnosis. HLH was considered given her hepatosplenomegaly, cytopenias, elevated CRP and normal ESR in the context of documented infection. Table 1 presents key HLH laboratory markers ordered at onset and their results. MAS, possibly associated with sJIA, was also considered despite the absence of arthritis, because of an intermittent, pink, net-like rash in the preceding weeks. Bone marrow evaluation was performed soon after her admission and demonstrated only rare hemophagocytes, similar to those seen in children with an underlying infection [5].

**Table 1:** Laboratory values meeting HLH diagnostic criteria found prior to, during and after trial of anakinra

**HLH diagnostic criteria**		**Patient laboratory values**		
Timing relative to anakinra trial		Admission to prior to anakinra	During anakinra (day from start of anakinra)	One week after anakinra
**Cytopenia in ≥ 2 cell lines**				
	Haemoglobin < 9g/dL (90g/L)	WNL	8.8 (d2); Nadir 5.2 (d16)	WNL
	Platelet < 100 × 10^9^/L	60	26 (d 16)	13-21
	Absolute neutrophil count < 1000 / μL	924	700 (d17)	48-153
**Hypertriglyceridemia and/or** **Hypofibrinogenemia**				
	Fasting triglyceride ≥ 265 mg/dL)	-	802 (d16)	423, 577, 726
	Fibrinogen < 150 mg/dL	127	WNL	-
**Ferritin ≥ 500 ng/mL (500 µg/L)**		905	629 (d15)	4814, 7597
**sCD25 ≥ 2400 μg/mL**		-	42,140 (d17)	-
**Decreased or absent NK cell activity**				
	LU_20_ NK ^a^	108	37.4 (d4)	-
	CD107a ^b^	-	7 % (d17)	-
**Hemophagocytosis in bone marrow**		“some”		“numerous”

-: laboratory value not measured; d: day from start of anakinra; NK: natural killer; WNL: within normal limits.

^a^ LU_20_ NK: Patient NK cells were cultured with radioisotope-labeled tumor cells. Functional NK cell activity measured by quantifying released isotope after correction for patient peripheral blood mononuclear cell % NK.

^b^ CD107a: Mononuclear cells from peripheral blood were stimulated with K562 target cells to induce degranulation. Six hours post incubation, CD107a expression was detected by flow cytometry.

**Table 2:** Laboratory values meeting HLH diagnostic criteria found prior to, during and after trial of anakinra

**HLH diagnostic criteria**		**Patient laboratory values**		
Timing relative to anakinra trial		Admission to prior to anakinra	During anakinra	One week after anakinra
**Cytopenia in ≥ 2 cell lines**				
	Anemia	No	Yes	No
	Thrombocytopenia	Yes	Yes	Yes
	Neutropenia	Yes	Yes	Yes
**Fasting Hypertriglyceridemia**		-	Yes	Yes
**Hypofibrinogenemia**		Yes	No	-
**Hyperferritinemia**		Yes	Yes	Yes
**Elevated sCD25**		-	Yes	-
**NK cell activity**				
	LU_20_ NK	reduced	markedly reduced	-
	CD107a	-	Yes	-
**Hemophagocytosis in bone marrow**		some	-	numerous

-: laboratory value not measured; NK: natural killer.

**Figure 1:**
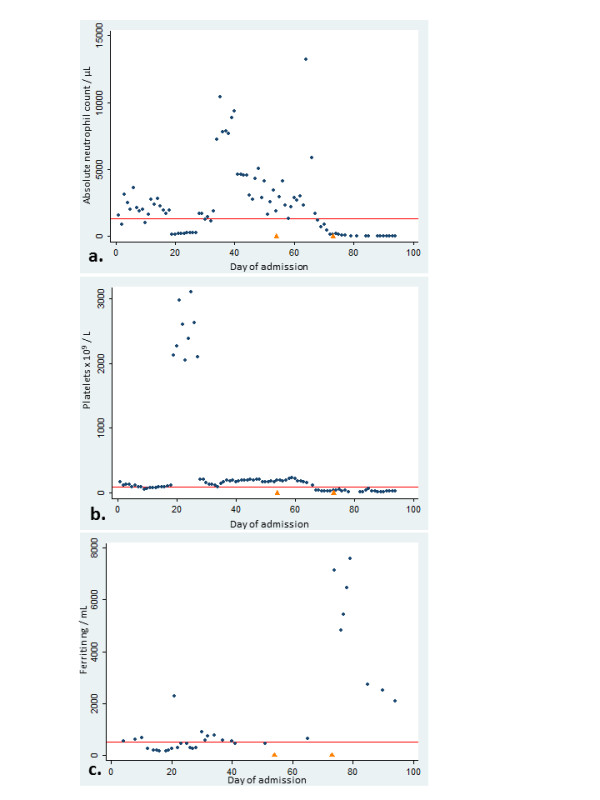
**Hemophagocyte in bone marrow. **Wright stain at 100x magnification. Macrophage with ingested nucleated red blood cells, nonnucleated erythrocyte and a lymphocyte. Aspirate was taken the day after discontinuation of anakinra.

Over the next month our patient continued to be dependent upon mechanical ventilation and developed acute respiratory distress syndrome (ARDS). Other complications included respiratory syncitial virus (RSV) infection and central line-related deep vein thrombosis requiring a six-week course of heparin sulfate. A brain MRI showed diffuse patchy white matter lesions most prominent in the right frontal lobe, suggestive of ischemia. Concern arose for adrenal insufficiency and she received several courses of stress dose corticosteroids with intermittent tapers. Intermittent episodes of fever and worsening cytopenias continued over the next month and HLH, possibly MAS, remained a consideration. For this reason an 18-day trial of anakinra was undertaken. However, fevers continued and abnormalities in liver enzymes along with hematologic abnormalities continued.

At this point, genetic testing results became available and identified two mutations in MUNC13-4: a known pathogenic mutation 1389(+1) G > A, a splice donor site of intron 15, and a second previously unidentified mutation, 1847 A > G. This second mutation was located in the splice donor site of exon 20, and the A > G change at this position would likely cause splicing error. It was unclear if this represented a compound heterozygous mutation or if the two variants were on a single chromosome. Parental testing was initiated. Given the genetic results and her persistent symptoms, the laboratory and bone marrow evaluations for HLH were reconsidered.

**Figure 2:**
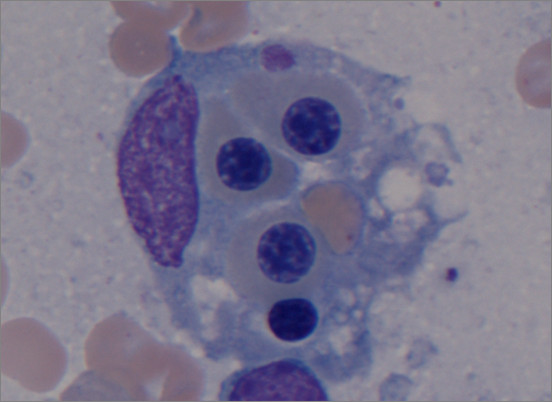
**Levels for absolute neutrophil count (a.), platelet count (b.) and ferritin (c.) in association with anakinra trial. **The horizontal axis in each figure indicates time in days from admission. The orange triangles indicate the first and last day that daily anakinra was given. In Figure 2a and 2b the red line denotes the threshold below which diagnostic criteria is met for HLH while in Figure 2c this line denotes the threshold above which HLH criteria is met.

Investigations relevant to HLH before, during and after anakinra use were evaluated (Tables 1 and 2). When compared to the initial assessment, there was subsequent marked reduction in natural killer (NK) cell function, CD107a upregulation and an elevation in soluble IL-2 (soluble CD25) receptor. Importantly, a drop in ANC (Figure 2a) and platelet count (Figure 2b) was identified during the end of the anakinra trial. Children with active HLH have been noted to have elevated white cell and platelet counts initially that decrease over time [6], as illustrated in our case. As a confounding feature, however, cytopenia is an uncommon but known side effect of anakinra. It is thus possible that addition of anakinra in our patient, with underlying MUNC 13–4 mutations, magnified the drop in platelet count and ANC that would have been seen with HLH alone. In addition, when ferritin was measured on the 15^th^ day of anakinra therapy, the level was 629 ng/mL (above the aforementioned acceptable limit of 500 ng/mL), whereas one day after anakinra treatment was terminated, the ferritin level increased to 7129 ng/mL (Figure 2c). IL-1 is known to increase the synthesis of ferritin subunits *in vitro*[7]. We hypothesize, therefore, that anakinra administration inhibited the surge of ferritin that would have otherwise been identified in association with clinical progression of HLH.

**Figure 3:**
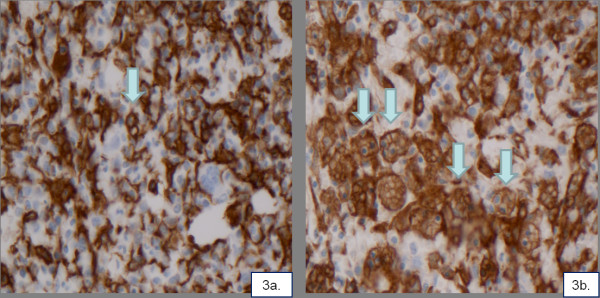
**Comparison of CD163 immunohistochemical stain of bone marrow one month before trial of anakinra trial (3a), and the day after discontinuation (3b). **Shown at 40x magnification. CD163 is an immunohistochemical stain that is specific for monocytes and macrophages and the positive cells show brown surface staining. Prior to anakinra, there are slit-like macrophages and some with a few ingested cells (blue arrow). On repeat biopsy and stain done after anakinra, there is an increase in the number of macrophages and the CD163 stain demonstrates numerous hemophagocytic macrophages containing multiple ingested cells (blue arrows).

A repeat bone marrow biopsy and the first and second bone biopsies were compared (Figure 2 and Figure 3). The first and second bone marrow biopsies were also critically compared (Figure 3). The latter showing a substantive increase in hematophagocytes compared to the former. In addition, a lumbar puncture showed an elevated protein level (109 mg/dL) with a lymphocytic pleocytosis (12 WBC with 100% lymphocytes). These findings suggested central nervous system HLH involvement.

Table  1: Initial laboratory values

Table  2: Evolution of laboratory values

Figure 1: Illustration of key laboratory value evolution

Figure 2: Bone marrow aspirate; Wright stain post-anakinra

Figure 3: CD 163 immunohistochemical stain of bone marrow biopsy

With this collective evidence, the HLH-2004 clinical protocol was initiated [4]. Our patient was treated with dexamethasone and etoposide. Cyclosporine was not initially utilized because of concern of abnormal kidney and liver function, but was added later as she improved.

Parental genetic testing was performed in order to determine if her case was likely primary HLH with familial genetic etiology. Testing confirmed that each parent carried one of the MUNC 13-4 mutations found in the patient. One parent possessed the novel variant identified while the other carried the known splice site mutation thus defining the patient as a compound UNC13D heterozygote consistent with FHL3.

Our patient was hospitalized for almost 4 months prior to transfer to a rehabilitation institution. She received a tracheostomy tube prior to discharge because of her inability to be weaned from respiratory support. She developed mild hypertension which was attributed to cyclosporine. Her liver enzymes continue to decrease although she has a persistently enlarged liver and spleen. She has not had any recent fevers and has an age-appropriate neurologic exam. However, cognitive limitations cannot be predicted. An unrelated HLA-matched donor has been identified and she will be proceeding to bone marrow transplant.

## Discussion

Hemophagocytic lymphohistiocytosis is a potentially fatal disease characterized by excessive macrophage and lymphocyte activity. The onset of HLH in a susceptible individual typically follows either a documented or presumed viral infection. Presently, HLH is often classified into primary (familial) and secondary (acquired) HLH.

The incidence of primary HLH is approximately 1:50,000 live born children [8]. However, HLH incidence varies in clinical studies, most likely due to a difference in prevalence across ethnic groups and/or emerging awareness [9]. Although HLH often presents between birth and 18 months of age, onset in older age groups is possible and has been shown to be a feature of particular gene mutations [10]. Familial HLH has a median survival of less than 2 months after diagnosis if it remains untreated [9]. It is likely that many cases are misdiagnosed as severe fatal infection.

In both primary and secondary HLH, Epstein Barr virus is the most commonly identified inciting infection, although cases associated with cytomegalovirus, human herpes virus 8, influenza, parvovirus, enterovirus and human immunodeficiency virus have also been described [11]. In secondary HLH, associated malignancies include neuroblastoma, Non-Hodgkin’s lymphoma and Langerhans’ cell histiocytosis.

Macrophage activation syndrome (MAS) can be considered a form of secondary HLH syndrome associated with autoimmune disease. MAS complicates an estimated 10% of Systemic Juvenile Idiopathic Arthritis (sJIA) cases [12], and approximately 1-5% of Systemic Lupus Erythematosus (SLE) cases [13]. Clinical features of MAS similar to primary HLH include high unremitting fever, hepatosplenomegaly, hepatic dysfunction, lymphadenopathy, encephalopathy, cytopenia, elevated ferritin and coagulopathy [12,14]. Laboratory similarities include depressed natural killer (NK) cell cytotoxic function, elevated soluble IL-2 receptor levels and soluble CD163. In its early stages, MAS can be a diagnostic challenge due to the overlap of symptoms with the underlying autoimmune disease. Factors potentially differentiating HLH from MAS include: 1.) change from quotidian to persistent fever pattern; 2.) sudden change from cytosis to cytopenia; 3.) coagulopathy; and 4.) decreasing ESR. Preliminary diagnostic criteria have been examined for MAS in sJIA, which may help to improve diagnosis of this condition in these patients [13,15].

As mentioned before, HLH is diagnosed through clear clinical criteria. Despite awareness of the diagnostic criteria, HLH diagnosis can be challenging because of the variability in presentation. Symptoms, especially if infection associated, may spontaneously remit [3]. Individual diagnostic criteria can be observed at distinct points in the disease course and can also remit and recur. Moreover, many of the criteria are non-specific for HLH, making it a diagnosis of exclusion in many cases. Finally, criteria such as NK cell function and genetic testing can take weeks to be finalized. Given all these challenges, it is important to consider HLH as a diagnosis both at onset and in the early stages of the disease process. In a review of familial and acquired HLH, key laboratory findings in establishing a diagnosis were identified as negative or decreased NK function as well as elevated soluble CD25 levels in all patients [16], suggesting these studies must be evaluated early on in the clinical course. In some centers, CD107a upregulation, a marker of NK cell degranulation is used as a surrogate for NK cell cytotoxicity [17]. However, as depressed NK cell cytotoxicity is seen in many conditions secondary to inflammation or immunosuppressive factors, other symptoms must be considered. Fever and splenomegaly occur in approximately 70% of HLH patients [16]. Fevers may be protracted, variable and may even resolve spontaneously. About 50% of patients initially present with elevated triglycerides, high ferritin, high LDH and/or a combination of anemia, neutropenia and thrombocytopenia [8,16]. Of note, ferritin is rarely > 200 μg/L in pediatric patients with infections outside of the context of HLH [16] and levels higher than this should raise suspicion for HLH. Lastly, hemophagocytosis is seen in less than 40% of patients at onset but is present in >80% of patients at time of diagnosis [16].

There are presently five types of familial HLH (FHL) [9], all of which impair the lytic activity of cytotoxic lymphocytes. Specifically, cytotoxic lymphocytes mediate contact-dependent elimination of cells perceived as dangerous by secreting preformed destructive molecules contained within specialized organelles termed lytic granules [2]. In order to mediate cytotoxicity, lytic granules must contain appropriate effector molecules and be localized to the contact site with the cell targeted for destruction. Once localized to that intercellular interface, the lytic granules dock at the cell membrane. The membrane of the granule and the cytotoxic lymphocyte are fused, allowing the release of the lytic effector molecules onto the targeted cell.

Familial HLH type 1 (FHL1) is linked to chromosome 9q21, but the exact gene remains unknown [9]. Perforin (PRF-1) was the first identified FHL gene (located on chromosome 10q21–22), and is responsible for familial HLH type 2 (FHL2).

Perforin protein is an effector molecule found in lytic granules. Once secreted, perforin inserts into the membrane of the target cell and facilitates the uptake of granzyme B and other cytolytic molecules contained in the lytic granules, into the target cell to induce cell death [1,9].

In perforin-deficient mice infected with high doses of lymphocytic choriomeningitis virus (LMCV), haemophagocytic lymphohistiocytosis could be induced, similar to human FHL [18]. This suggests that failure to clear virus will lead to persistence of viral antigens and prolonged CD8 T cell activation and cytokine production. Other animal model studies demonstrate perforin’s role in regulating lymphocyte number in autoimmunity [19], after microbial infection [20] and when other cell-death pathways are impaired [21]. The extent that these mechanisms are involved in the control of immune responses, however, is still speculative.

PRF-1 mutations account for approximately 20-40% of FHL cases. It is important to note however that CD107a up-regulation is usually normal in FHL2 as CD107a represents a measure of degranulation [22].

FHL3 is caused by mutations in the UNC13D gene located on chromosome 17q25. UNC13D encodes the protein MUNC 13–4. Found on the surface of lytic granules, MUNC 13–4 is required for priming the lytic granules for docking at the cytotoxic cell membrane [9,12]. FHL 4 is caused by mutations in STX11 on chromosome 6q24 that encodes the syntaxin 11 protein. Syntaxin-11 is a member of the SNARE protein family and facilitates the fusion of the lytic granule membrane with that of the cytotoxic lymphocyte [1,23]. FHL5 is caused by mutations in MUNC 18–2 located on chromosome 19p13. MUNC 18–2 encodes the syntaxin binding protein 2. It is a partner of syntaxin 11 and is required for SNARE complex-mediated fusion of the lytic granule with the cytolytic cell membrane [9,22].

In addition to genetic defects associated with FHL, there are also immunodeficiency syndromes associated with HLH that impair the secretion of lytic granule contents. These include Griscelli syndrome type 2, Hermansky Pudlak type II, Chediak Higashi and X-linked lymphoproliferative disease Type 1. Griscelli syndrome type 2 and Chediak Higashi syndrome are typically associated with albinism due to an effect upon melanocyte pigment secretion. Griscelli syndrome type 2 is identified by mutations in RAB27A while Chediak Higashi syndrome is associated with LYST mutations. Mutations of both these gene impair proteins important for formation and/or trafficking of secretory lysosomes [24]. In these syndromes, a gene defect interferes with lytic granules reaching the cytotoxic lymphocyte membrane, thus leading to impaired NK cell cytotoxicity. Patients with X-linked lymphoproliferative disease have difficulty clearing Epstein-Barr virus (EBV) infected B-cells, with subsequent extensive lymphocytic expansion into multiple organs. The SH2D1A mutation, which encodes for the signaling lymphocyte activation molecule (SLAM)-associated protein SAP, is identified in XLP1. Impaired cytolytic function in XLP1 is thought to cause accumulation EBV-infected B cells and persistence of reactive inflammatory cells, which combine to produce an exaggerated immune response [25].

Treatment for HLH is described in the HLH 2004 revised guidelines [14] and is divided into acute and long-term management. Initial treatment with an immunomodulatory regimen is recommended. Patients with primary HLH who fail to reach disease resolution within 8 weeks of treatment should continue on this regimen for an additional treatment course. Hematopoetic stem cell transplantation should be pursued as soon as a suitable donor is available for all patients with primary HLH, relapsed HLH, or those failing to progress on therapy [4]. There is no standard for the treatment of secondary HLH or MAS in the context of rheumatologic disease. High dose corticosteroids, biologic agents, cyclosporine and high dose intravenous immunoglobulin have been used with varying success. Anakinra, a recombinant IL-1 receptor antagonist, has been increasingly used for sJIA patients with MAS [24]. If initial treatment fails, HLH salvage therapy may be pursued according to the HLH 2004 chemotherapeutic regimens [14,12]. Confirmation of a genetic mutation is not needed for immediate management but is important in differentiating familial HLH and secondary HLH for long-term patient management and family genetic counseling.

While anakinra has been successfully utilized in MAS, it has not been formally studied in HLH associated with bonafide mutations. In our patient with two identified UNC13D mutations, anakinra was utilized as an immunomodulator while the diagnosis was evolving. Although the progression of our patient’s disease was not florid while receiving anakinra, there were decreased platelet and neutrophil counts during therapy. However, when anakinra was stopped, there was a clear surge in ferritin levels. It is unclear from our experience if anakinra magnifies certain abnormalities found in primary HLH while moderately quelling others. A more likely explanation is that the anakinra functioned in partially blocking the HLH-associated inflammation in our patient thus allowing for the masking of certain phenotypic associations of HLH such as ferritin, but not others (i.e., platelet counts). While we were not situated to immunologically prove the partial blockade of inflammation in our patient while receiving anakinra, our experience suggests that its use be reserved for the more mild secondary forms of HLH and that therapy of primary HLH be limited to more wide-ranging immunosuppression such as that provided through the HLH-2004 protocol [4,14].

## Conclusion

HLH is a clinical syndrome that remains difficult to diagnose. Our patient’s case demonstrates that use of immunosuppressive agents can cloud diagnosis. As clinicians, it is important to be aware of this in order to avoid delay in diagnosis and life-saving therapy.

## Consent

“Written informed consent was obtained from the parent of the patient for publication of this Case Report and any accompanying images. A copy of the written consent is available for review by the Editor-in-Chief of this journal.”

## Abbreviations

ARDS: Acute respiratory distress syndrome; FHL: Familial hematophagocytic lymphohystiocytosis; HLH: Hematophagocytic lymphohistiocytosis; IL-1: Interleukin 1; MAS: Macrophage Activation Syndrome; NK: Natural killer; RSV: Respiratory Syncitial Virus; SJIA: Systemic Juvenile Idiopathic Arthritis; XLP: X-Linked lymphoproliferative syndrome.

## Competing interests

The following authors certify that they have no competing interest: Jackie Garrett, Irene Fung, Jeremy Rupon, Andrea Knight, Michelle Paessler, Melissa Mizesko, Jordan Orange

## Authors’ contributions

JG: wrote the initial manuscript draft and helped with initial revisions; IF: reviewed patient’s data, provided tables and figures along with interpretation, and made initial revisions; JR: assisted in caring for the patient, making diagnosis and is involved in follow-up care for patient; AK assisted in caring for the patient, making the diagnosis and made initial revisions; MM: assisted in caring for the patient and making the diagnosis; MP developed the CD163 immunohistochemical stain for use at our institution. JO: assisted in caring for the patient, making the diagnosis and supervised the research; JG and IF share in first author status. All authors read and approved the final manuscript.

## Authors’ information

JG: Fellow, Division of Allergy and Immunology, Children’s Hospital of Philadelphia; Clinical Instructor, Department of Pediatrics, The Perelman School of Medicine at the University of Pennsylvania.

IF: Fellow, Division of Allergy and Immunology, Children’s Hospital of Philadelphia.

JR: Fellow, Division of Hematology and Oncology, Children’s Hospital of Philadelphia.

AK: Fellow, Division of Rheumatology, Children’s Hospital of Philadelphia.

MM: Fellow, Division of Rheumatology, Children’s Hospital of Philadelphia.

MP: Assistant Professor, Department of Pathology and Laboratory Medicine, Children’s Hospital of Philadelphia, University of Pennsylvania School of Medicine.

JO: Associate Professor, Department of Pediatrics, Children’s Hospital of Philadelphia, University of Pennsylvania School of Medicine.
